# Invasive Coronary Physiology in Contemporary Practice: From Lesion Selection to Comprehensive PCI Guidance and Functional Phenotyping

**DOI:** 10.3390/jcm15134915

**Published:** 2026-06-24

**Authors:** Francesco Maria Sparasci, Luca Raone, Mario Iannaccone, Cosmo Godino, Alessandro Mandurino-Mirizzi

**Affiliations:** 1 Department of Molecular Medicine, University of Pavia, 27100 Pavia, Italy; sparasci.francesco@gmail.com (F.M.S.); raone.luca@gmail.com (L.R.); 2 S.G. Bosco Turin Nord Emergency Hospital, ASL Città di Torino, 10154 Turin, Italy; mario.iannaccone@hotmail.it; 3 Cardiology Unit, Heart Valve Center, IRCCS San Raffaele Scientific Institute, 20132 Milan, Italy; cosmogodino@gmail.com; 4 Faculty of Medicine, Vita-Salute San Raffaele University, 20132 Milan, Italy; 5 Division of Cardiology, “Vito Fazzi” Hospital, 73100 Lecce, Italy; 6 Department of Experimental Medicine (DiMeS), University of Salento, 73100 Lecce, Italy

**Keywords:** coronary physiology, fractional flow reserve, non-hyperemic pressure ratios, pullback pressure gradient, percutaneous coronary intervention, coronary microvascular dysfunction, ANOCA, INOCA

## Abstract

**Background/Objectives**: Invasive coronary physiology has evolved from a tool for assessing intermediate stenoses to a comprehensive framework for guiding diagnosis and treatment across the spectrum of coronary artery disease (CAD). This review aims to provide an updated, catheterization laboratory-centered overview of contemporary invasive coronary physiology, emphasizing its role in optimizing percutaneous coronary intervention (PCI) and in evaluating patients with angina and non-obstructive coronary arteries (ANOCA/INOCA). **Methods**: A narrative review of contemporary evidence, including randomized trials, consensus documents, and guideline recommendations, was conducted. Key physiological indices—fractional flow reserve (FFR), non-hyperemic pressure ratios (NHPRs), coronary flow reserve (CFR), and index of microcirculatory resistance (IMR)—were examined alongside emerging tools such as longitudinal vessel analysis and the pullback pressure gradient (PPG). Applications in pre- and post-PCI assessment, physiology–imaging integration, and comprehensive functional testing in ANOCA/INOCA were evaluated. **Results**: Physiology-guided PCI improves clinical outcomes and resource utilization compared with angiography-guided strategies. Longitudinal vessel assessment and PPG enable characterization of focal versus diffuse CAD, improving procedural planning and prediction of post-PCI physiological results. Post-PCI physiological assessment identifies residual ischemia and guides optimization strategies. In patients without obstructive CAD, combined assessment of microvascular function and vasomotor reactivity allows identification of distinct pathophysiological endotypes, supporting mechanism-based, individualized therapy. Integration with intracoronary imaging further enhances procedural precision. **Conclusions**: Contemporary invasive coronary physiology provides a multidimensional approach integrating epicardial, microvascular, and vasomotor domains. This framework supports personalized decision-making, optimizes revascularization, and reduces unnecessary interventions, representing a cornerstone of modern coronary care.

## 1. Introduction

Invasive coronary physiology has gained increasing importance in catheterization laboratories over recent decades. Angiographic assessment of coronary artery disease (CAD) should be integrated with physiological measurements to ensure appropriate management of intermediate coronary stenoses, thereby avoiding the treatment of functionally non-significant lesions [1,2]. The management of CAD has progressively shifted toward an established physiology-guided PCI approach, supported by randomized controlled trials demonstrating a reduction in major adverse cardiovascular events compared with optimal medical therapy alone [3,4].

As coronary physiology has become established in PCI guidelines, several studies have also demonstrated its possible role in optimizing revascularization. In particular, there is growing evidence that suboptimal physiology after PCI is associated with worse clinical outcomes [5,6,7].

Emerging physiological data suggest that CAD encompasses different phenotypes, not all of which respond favorably to conventional PCI with stent implantation. For example, in the presence of diffuse CAD, PCI with stent implantation often fails to achieve a satisfactory reduction in trans-lesional pressure gradient, probably due to the extent and characteristics of the disease. Consequently, the pullback technique for longitudinal vessel assessment has become pivotal in planning PCI and predicting post-procedural physiological outcomes [8,9]. Pullback analysis can be performed using either non-hyperemic pressure ratios (NHPRs) or hyperemic indices such as fractional flow reserve (FFR), underscoring the versatility and clinical relevance of this step in physiology-guided PCI [10]. More recently, attention has focused on the interpretation of pullback data through a dedicated index—the pullback pressure gradient (PPG)—which aims to quantify the distribution of CAD along the vessel, ranging from focal to diffuse disease patterns [11].

Another important application of invasive coronary physiology lies in the evaluation of angina or ischemia with no obstructive coronary arteries (ANOCA/INOCA). A substantial proportion of patients undergoing coronary angiography (approximately 40–70%) show no significant epicardial CAD, and physiological assessment is essential to identify underlying mechanisms of ischemia. Similar to obstructive CAD, ANOCA/INOCA encompasses multiple pathophysiological phenotypes, each requiring a tailored therapeutic approach. Comprehensive evaluation should therefore include assessment of the coronary microcirculation as well as vasoreactivity testing [1,2]. Such an approach enables physiology-guided medical therapy, reducing reliance on empiric treatment strategies and improving patient understanding of their condition.

Contemporary invasive coronary physiology should be viewed not merely as a lesion-selection tool, but as a comprehensive decision-making framework integrating epicardial, microvascular, and vasomotor domains. Within this perspective, this review aims to provide an updated overview of invasive coronary physiology during coronary angiography from a catheterization laboratory-centered perspective, with a primary focus on chronic coronary syndromes, physiology-guided PCI planning and optimization, and invasive functional assessment in patients with ANOCA/INOCA.

## 2. Materials and Methods

This manuscript is a narrative review providing an updated overview of invasive coronary physiology in the catheterization laboratory. A structured literature search was performed using PubMed/MEDLINE, Embase, and the Cochrane Library for studies published up to April 2026. Search terms included combinations of fractional flow reserve, non-hyperemic pressure ratios, instantaneous wave-free ratio, pullback analysis, pullback pressure gradient, percutaneous coronary intervention, coronary microvascular dysfunction, coronary flow reserve, index of microcirculatory resistance, vasoreactivity testing, ANOCA, and INOCA. Reference lists of relevant articles were also screened to identify additional studies.

Eligible studies included randomized controlled trials, observational studies, and consensus documents or guidelines addressing invasive coronary physiology and physiology-guided management. Emphasis was placed on high-quality and contemporary evidence. Data were qualitatively synthesized across key domains, including epicardial physiological assessment, longitudinal vessel analysis, PCI optimization strategies, physiology–imaging integration, and invasive evaluation of microvascular and vasomotor disorders. No quantitative meta-analysis was performed.

The PubMed search strategy yielded approximately 1600 records up to April 2026. Similar searches were performed in Embase and the Cochrane Library. Records were assessed for relevance to the predefined topics of this narrative review, and the most influential contemporary studies, randomized trials, guideline documents, consensus statements, and landmark investigations were selected for qualitative synthesis. A total of 70 references were included in the final manuscript.

This narrative review was not conducted according to a systematic review methodology and therefore did not include a formal study selection process, quality assessment, or quantitative synthesis of the available evidence. As with all narrative reviews, the selection of the cited literature may be influenced by publication bias and the predominance of positive findings reported in the available studies, including landmark randomized clinical trials. In addition, several studies in the field of coronary physiology have enrolled predominantly male populations, limiting the availability of sex-disaggregated data and potentially affecting the generalizability of some findings to women.

## 3. Results and Discussion

### 3.1. Functional Assessment of Epicardial CAD

Traditional invasive coronary physiology was originally developed to determine the hemodynamic significance of angiographically intermediate epicardial stenoses, conventionally defined as lesions causing a 40–70% diameter reduction, or up to 90% when angiographic severity is uncertain [1,2]. By recording intracoronary pressure under conditions of maximal hyperemia—when a near-linear relationship between pressure and flow can be assumed—fractional flow reserve (FFR) enables lesion-specific assessment of ischemic potential [8]. A large body of evidence has demonstrated that FFR-guided PCI simplifies interventional strategies, reduces the number of treated vessels and implanted stents, lowers rates of major adverse cardiovascular events, and represents a cost-effective approach for healthcare systems [3,4,12,13,14,15].

To overcome the need for pharmacological hyperemia, non-hyperemic pressure ratios (NHPRs) were subsequently developed. Among these, the instantaneous wave-free ratio (iFR) was the first resting index shown to be non-inferior to FFR for PCI guidance in large randomized trials [16,17]. Although longer-term follow-up has suggested numerically lower event rates with FFR-guided strategies, contemporary ESC guidelines place FFR and iFR on equal footing, granting both strong recommendations for clinical decision-making [1,2,18,19]. Additional NHPRs, including diastolic pressure ratio (dPR) and resting full-cycle ratio (RFR), have further expanded the portfolio of resting indices available for physiological assessment. Although large outcome-driven randomized trials are lacking for these newer indices, their strong correlation with both FFR and iFR supports the concept of a class effect among NHPRs [20].

Despite the overall concordance between hyperemic and non-hyperemic indices, discordance between FFR and NHPRs is observed in approximately 15–20% of intermediate coronary lesions [21]. This phenomenon reflects fundamental physiological differences, as FFR is inherently flow-dependent and influenced by hyperemic microvascular resistance, whereas NHPRs are measured under resting conditions and may be more sensitive to baseline flow variations [22]. Discordant patterns—such as abnormal FFR with normal NHPRs or vice versa—are more frequently observed in the presence of diffuse atherosclerosis, microvascular dysfunction, or borderline stenoses [23]. From a clinical perspective, these scenarios remain challenging, as outcome data are less robust and optimal management is not fully established. In such cases, integration of clinical presentation, lesion morphology, and adjunctive physiological or imaging data may help guide decision-making, rather than reliance on a single index alone [16].

#### 3.1.1. Pre-PCI Functional Assessment: Clinical Value

**Figure 1:**
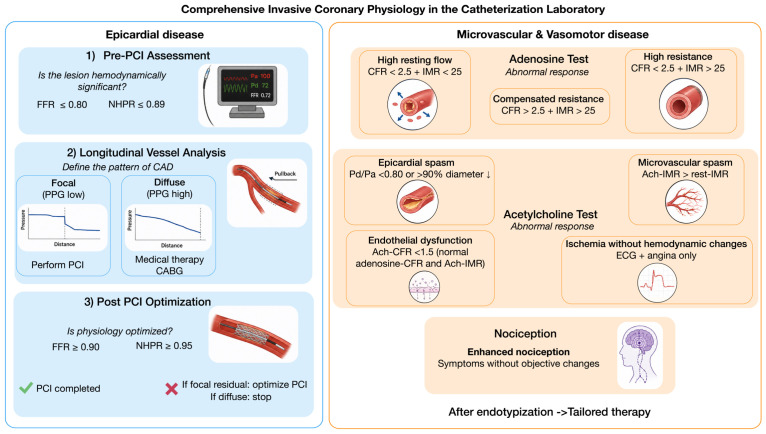
A comprehensive, physiology-guided framework integrating epicardial, vasomotor, and microvascular assessment to enable precision management of coronary artery disease. PCI: Percutaneous Coronary Intervention, FFR: Fractional Flow Reserve, NHPR: Non-Hyperemic Pressure Ratio, CFR: Coronary Flow Reserve, IMR: Index of Microcirculatory Resistance, Pd/Pa: Distal Coronary Pressure to Aortic Pressure Ratio, Ach: Acetylcholine, ECG: electrocardiogram, CAD: Coronary Artery Disease.

Planning of PCI is a pivotal step in confirming the need for revascularization and in predicting the likelihood of achieving an optimal result (Figure 1). In this setting, the 2024 ESC Guidelines on chronic coronary syndromes (CCS) underscore the central role of a physiology-based approach in the management of stable CAD. In patients with angiographically intermediate coronary stenoses, functional assessment with FFR or NHPRs—including iFR and RFR—is recommended with a Class I, Level of Evidence A indication [1]. This position closely aligns with contemporary AHA/ACC guidance for CCS, with both societies converging on physiology-guided lesion selection as the standard of care [2]. Beyond single-vessel disease, invasive coronary physiology plays a key role in guiding revascularization strategies in patients with multivessel CAD [1].

Pre-PCI physiological assessment provides several advantages to the interventionalist. Beyond single-point measurements of distal pressure gradients, longitudinal vessel analysis adds information on the functional distribution of coronary disease. This approach helps determine the indication for PCI, identify patterns of flow-limiting disease, simulate the physiological impact of stenting at specific locations, and facilitate precision stent deployment [10].

Despite the robust evidence supporting physiology-guided decision-making in CCS, important knowledge gaps remain in specific clinical settings. In patients with severe aortic stenosis, left ventricular hypertrophy, microvascular dysfunction, and altered coronary hemodynamics may influence the interpretation of invasive physiological indices, and the optimal integration of coronary physiology into revascularization strategies remains an area of ongoing investigation [24]. Similarly, in acute coronary syndromes, functional assessment has been shown to improve clinical outcomes, particularly by reducing repeat revascularizations; however, available studies have yielded heterogeneous results, and the superiority of physiology-guided strategies over angiography-guided approaches has not been consistently demonstrated [25,26].

#### 3.1.2. Pre-PCI Functional Assessment: Longitudinal Vessel Analysis

By mapping pressure gradients along the entire vessel during pressure-wire pullback, longitudinal vessel analysis overcomes the inherent limitations of single-point FFR or NHPR measurements and enables lesion-specific physiological assessment.

The clinical importance of comprehensive physiological assessment prior to PCI was highlighted in the DEFINE-PCI trial. In this study, vessels with angiographic stenosis ≥ 40% and positive NHPR (iFR < 0.90) underwent angiography-guided PCI without systematic baseline pullback analysis. Despite angiographically successful revascularization, residual ischemia, defined as a distal vessel iFR < 0.90 after PCI, was observed in 21.9% of vessels and 24% of patients, suggesting that reliance on angiographic guidance alone may result in incomplete physiological revascularization [5,6]. These findings support the role of pre-PCI pullback analysis in improving procedural planning and optimizing physiological outcomes.

For global vessel evaluation, the pressure sensor should be positioned in the distal third of the vessel—typically at least 20–30 mm beyond the target lesion—and a gradual pullback performed to characterize the distribution of pressure loss along the coronary artery [10,27]. Integration of longitudinal physiological data with coronary angiography further enhances spatial co-localization of hemodynamically significant disease. Co-registration technologies generate physiological maps that depict the distribution of pressure loss along the vessel. This approach is particularly useful in vessels with tandem lesions or diffuse disease, where angiographic assessment alone may underestimate the complexity of physiological impairment. In this context, longitudinal physiological mapping has contributed to the development of the concept of physiology-guided “virtual PCI”, which enables estimation of the expected physiological improvement following lesion treatment [27,28,29].

Longitudinal physiological assessment also enables characterization of distinct phenotypes of epicardial CAD, including focal pressure gradients, serial stenoses, and diffuse atherosclerotic disease. Reflecting the clinical relevance of these patterns, the 2023 EAPCI consensus document proposed standardized definitions for pre-PCI physiological disease patterns based on pullback analysis (Table 1) [10]. This classification provides a basis for tailoring revascularization strategies: when pullback analysis predicts an optimal or acceptable physiological result, PCI can proceed as planned; conversely, if a suboptimal post-intervention physiological outcome is anticipated, alternative strategies—including coronary artery bypass grafting or optimal medical therapy—should be considered.

Nevertheless, the prognostic implications of different physiological disease patterns and the optimal management of vessels with diffuse physiological impairment remain incompletely defined. This issue may be particularly relevant in the left anterior descending artery (LAD), where diffuse physiological abnormalities are common and may reflect vessel-specific anatomical and hemodynamic characteristics. Emerging data suggest that patients with diffusely positive iFR patterns in the LAD who are managed conservatively may experience long-term outcomes comparable to those of patients with negative iFR values, highlighting the need for further studies to better define the prognostic significance and therapeutic implications of diffuse physiological disease in this vessel [30].

#### 3.1.3. Pre-PCI Functional Assessment: Pullback Pressure Gradient

Despite increasing recognition of longitudinal coronary physiology, interpretation of pressure pullback tracings remains largely qualitative, operator-dependent, and poorly standardized. The PPG has emerged as a novel index designed to address these limitations by providing a quantitative and reproducible characterization of the longitudinal distribution of epicardial resistance [11]. PPG is derived from two components: (1) the largest FFR gradient observed during pullback and (2) the overall extent of functional disease along the vessel. In the original formulation using motorized hyperemic pullbacks, the equation was defined as:

(1) $$PPG\;index=\;\left\{\frac{{MaxPPG}_{20mm}}{{∆FFR}_{vessel}}+\left(1-\frac{{lenght\;of\;functional\;disease}_{\left(mm\right)}}{{total\;vessel\;lenght}_{\left(mm\right)}}\right)\right\}÷2\;$$

where *MaxPPG*_20*mm*_ represents the maximum FFR change over a 20 mm segment, Δ*FFR_vessel_* is the difference between distal and ostial FFR, and functional disease length corresponds to vessel segments with an FFR drop ≥ 0.0015 per mm. Because motorized pullback systems are not widely available and may prolong procedures, the index was subsequently adapted for manual pullbacks. In this simplified approach, spatial measurements are replaced by temporal measurements derived from the pullback duration. Accordingly, *MaxPPG* is redefined as the maximum FFR change occurring within 20% of the total pullback time, while Δ*FFR_vessel_* remains the difference between distal and ostial FFR. The resulting formula can be expressed as:

(2) $$PPG\;index=\left\{\frac{{MaxPPG}_{\;20\%\;time}}{{∆FFR}_{vessel}}+\left(1-\frac{{time\;with\;functional\;disease}_{\left(s\right)}}{{total\;vessel\;time}_{\left(s\right)}}\right)\right\}÷2$$

This temporal adaptation allows PPG calculation during routine manual hyperemic pullbacks, without dependence on the absolute pullback duration and with excellent reproducibility compared with motorized techniques [31].

Higher PPG values reflect a more focal pressure drop along the vessel, whereas lower values indicate a more diffuse pattern of disease. Accordingly, PPG should be interpreted as a continuous physiological measure rather than dichotomized using arbitrary thresholds. Consistent with this, cutoffs used to distinguish focal from diffuse disease have varied across studies (approximately 0.62 to 0.78), underscoring that PPG is best viewed as a continuous descriptor of the functional distribution of coronary disease [11,32].

Across studies within the Precise PCI Plan (P3) program, PPG has consistently differentiated focal from diffuse disease patterns and improved physiological interpretation beyond ischemic severity alone [11,33]. Higher PPG values have been associated with greater achievable physiological gain, higher post-PCI FFR, larger minimal stent area, and more favorable procedural characteristics. Conversely, low PPG values have been linked to limited functional improvement, higher rates of stent malapposition, and increased periprocedural myocardial injury [34,35]. Therefore, higher PPG values are associated with a greater likelihood that PCI may provide benefit; lower PPG values may be associated with limited benefit from revascularization.

In addition to conventional trans-lesional gradients, PPG provides complementary information and predicts the physiological benefit of PCI more accurately than other longitudinal assessment methods. In serial or mixed disease patterns, reliance on ΔFFR or ΔiFR alone may overestimate the benefit of focal intervention, as hyperemic flow through each stenosis is constrained by adjacent segments. As shown in prior studies, this interaction leads to overestimation of post-PCI physiological improvement in serial disease [36,37]. PPG may mitigate this limitation by characterizing resistance along the entire vessel, enabling more realistic prediction of post-PCI physiology [38,39]. Beyond procedural metrics, higher PPG values have been associated with greater angina relief and improved quality of life after PCI, while diffuse phenotypes derive less symptomatic benefit [38,39].

From a practical perspective, PPG may represent a valuable adjunct whenever invasive physiological assessment and longitudinal pullback analysis are performed, as it provides additional information on the functional pattern of coronary disease without requiring extra procedural steps or disposable equipment. However, several limitations should be acknowledged. Long-term outcome data remain limited, no randomized studies have evaluated PPG-guided revascularization strategies, and the optimal management of patients with low PPG values remains uncertain. Wider adoption may therefore depend not only on broader availability of dedicated software solutions and integration into routine physiological workflows, but also on further clinical validation and outcome-driven evidence.

#### 3.1.4. Post-PCI Functional Assessment: Clinical Value

Physiological assessment after angiographically satisfactory PCI allows early identification of residual flow-limiting disease. According to the 2024 ESC guidelines on CCS, post-PCI evaluation with FFR or NHPRs may identify patients at increased risk of persistent angina or adverse clinical outcomes (Class IIa, Level of Evidence B). Consistently, suboptimal post-PCI FFR values have been independently associated with worse prognosis, underscoring the relevance of physiological optimization beyond angiographic endpoints (Class IIb, Level of Evidence B) [1].

In addition to risk stratification, post-PCI physiological assessment identifies targets for further optimization and evaluates the impact of corrective interventions [10]. Within this approach, longitudinal vessel analysis enables identification of mechanisms underlying suboptimal functional results and informs the feasibility and strategy of further optimization. Following physiology-based optimization, repeat measurements may reveal residual disease not amenable to PCI, thereby guiding intensification of medical therapy (Figure 1) [1,10].

#### 3.1.5. Post-PCI Functional Assessment: Rationale

Post-PCI optimization without pre-procedural physiological assessment may be inconsistent. In the TARGET-FFR trial, over two-thirds of patients had suboptimal physiology after angiography-guided PCI, and FFR-guided optimization did not significantly increase the proportion achieving final FFR ≥ 0.90, although it reduced the rate of FFR < 0.80. Optimization was deferred in 25% of patients due to diffuse disease—patients who could likely have been identified by pre-PCI assessment [7].

Accordingly, whether post-PCI physiological assessment should be performed routinely or reserved for selected complex cases remains an area of ongoing debate. In the EASY-PREDICT trial, no significant clinical advantage was observed compared with angiography-guided PCI, although selected patient subsets may still derive benefit from a physiology-guided optimization strategy. Notably, the optimization target in this study was FFR > 0.80, which may have limited its ability to demonstrate a clinical benefit [40].

Routine post-PCI physiology is therefore unlikely to be universally beneficial. Its clinical value appears greatest in complex PCI, long or multivessel disease, and angiographically ambiguous results. Conversely, in focal lesions treated with optimal angiographic and intravascular imaging guidance, routine physiological reassessment may provide limited incremental benefit. Moreover, in diffuse disease patterns, persistently suboptimal post-PCI physiology may reflect a true physiological ceiling rather than a modifiable target, limiting the potential benefit of additional stenting [7,40,41].

#### 3.1.6. Post-PCI Functional Assessment: Optimization Strategies

Following stent implantation, post-procedural physiological reassessment provides an objective measure of procedural success. Optimal values allow safe termination of the procedure, whereas suboptimal physiology—commonly defined as FFR < 0.90 or NHPR < 0.95—should prompt structured repeat pullback analysis [5,6].

According to EAPCI definitions, post-PCI longitudinal vessel analysis characterizes residual disease into distinct phenotypes based on the distribution of residual pressure loss (Table 1) [10]. Not all suboptimal results mandate further mechanical intervention. Specifically, in the presence of focal pressure loss—whether in-stent or at proximal or distal segments—PCI optimization may improve the final physiological result. In contrast, when residual pressure loss is predominantly diffuse, additional stenting is unlikely to confer benefit and may increase procedural risk; in such cases, optimized medical therapy is preferred [10].

Moreover, particular caution is warranted when interpreting post-PCI physiological indices in the LAD. Compared with other coronary vessels, the LAD more frequently exhibits diffuse atherosclerotic disease and subtends a larger myocardial territory, resulting in lower post-PCI physiological values despite angiographically satisfactory PCI and optimal stent deployment [7]. Consistently, observational studies have demonstrated lower post-PCI FFR values and lower vessel-specific prognostic thresholds in LAD lesions compared with non-LAD vessels [42]. Therefore, failure to achieve commonly proposed post-PCI targets should not automatically be interpreted as procedural failure in LAD interventions [43].

#### 3.1.7. Physiology–Imaging Integration in PCI Guidance

Invasive coronary physiology can be integrated with angiography and/or intracoronary imaging, providing enhanced spatial information for both pre-PCI planning and post-PCI optimization.

Integration of physiological information on the presence and spatial distribution of flow-limiting stenoses with imaging-derived data on plaque morphology, plaque distribution, and vessel dimensions changes procedural planning, including lesion preparation, identification of appropriate plaque-free landing zones, and selection of optimal stent diameter and length. Multimodality co-registration combining coronary physiology, intracoronary imaging, and angiography is currently feasible with IVUS and iFR. Although angiographic co-registration is also available for OCT, imaging-derived physiological indices (e.g., OCT-based FFR) remain investigational and have not yet been validated for routine clinical use [10].

In addition, a substantial body of evidence indicates that PCI optimization guided by intravascular imaging is associated with improved long-term outcomes [7,44,45,46]. Notably, these benefits appear largely independent of the relatively modest increases in post-PCI FFR observed in imaging-guided optimization studies [44,47,48]. The combined use of intracoronary imaging and post-PCI longitudinal pressure pullback enables identification of the mechanisms underlying residual focal pressure gradients and facilitates targeted corrective strategies [48]. Furthermore, intracoronary imaging allows detection of high-risk morphological features of coronary disease—both before and after PCI—with prognostic relevance, even in the absence of inducible ischemia, such as stent malapposition or significant edge dissection [49].

Coronary physiology and intracoronary imaging should be regarded as complementary and integrated modalities for PCI guidance, each providing distinct but synergistic information. Physiological assessment, particularly when combined with longitudinal vessel analysis, enables the identification and spatial localization of hemodynamically relevant residual disease and supports procedural planning and optimization. Intravascular imaging allows direct characterization of the underlying mechanisms of residual stenosis, including stent underexpansion, edge dissection, and plaque-related constraints, thereby informing targeted corrective strategies. Accordingly, imaging and physiology should be considered as integrated tools across the PCI workflow, with imaging being particularly valuable in cases of suboptimal physiological or angiographic results after initial optimization.

However, the optimal sequencing and integration of physiology and intracoronary imaging remain incompletely defined, and whether systematic multimodality guidance provides incremental benefit over a selective approach requires further investigation.

Beyond this integrated paradigm, emerging developments in coronary physiology are increasingly moving toward simplified and wire-free approaches, including angiography-derived fractional flow reserve, computational hemodynamic modelling, and artificial intelligence-based reconstructions of coronary physiology. These technologies aim to extend functional assessment beyond the catheterization laboratory, potentially enabling broader lesion evaluation without pressure wire instrumentation. Although promising, their clinical adoption will depend on further validation in prospective outcome-driven studies and on their integration with established wire-based and imaging-guided strategies.

### 3.2. Functional Evaluation of Non-Obstructive Arteries

Beyond epicardial disease, invasive coronary physiology should be viewed as a comprehensive framework integrating epicardial, microvascular, and vasomotor domains, particularly in patients without obstructive coronary arteries. A substantial proportion of patients undergoing coronary angiography for angina or objective ischemia—up to 40–70% and 20–30%, respectively—do not exhibit flow-limiting epicardial CAD [50,51]. In these patients, mechanisms such as coronary microvascular dysfunction (CMD), vasomotor abnormalities, and myocardial bridging frequently underlie symptoms and often remain undetectable by angiography alone, supporting the role of invasive physiology-based diagnostic strategies [52,53,54].

Within the CCS spectrum, the 2024 ESC Guidelines further define ANOCA and INOCA as distinct clinical entities. In persistently symptomatic patients, invasive coronary functional testing—including coronary flow reserve (CFR), index of microcirculatory resistance (IMR), and acetylcholine testing—is endorsed to identify treatable endotypes and guide personalized therapy. This recommendation is supported by randomized evidence from the CorMicA trial, which demonstrated significant improvements in anginal symptoms and quality of life with a stratified, physiology-guided approach [55].

Moreover, in myocardial infarction with non-obstructive coronary arteries (MINOCA), when angiography alone fails to identify the underlying mechanism, both ESC guidance and the AHA Scientific Statement endorse a structured diagnostic pathway incorporating additional invasive evaluation—including left ventriculography, intracoronary physiological assessment of microvascular function and coronary reactivity, and intravascular imaging—to elucidate the pathophysiological substrate [56].

#### 3.2.1. CMD and Vasomotor Disorders

Invasive evaluation of the coronary microcirculation relies on complementary indices derived from thermodilution- or Doppler-based techniques. CFR reflects the integrated capacity of the coronary circulation to augment flow in response to increased myocardial demand, encompassing both epicardial and microvascular contributions, whereas IMR provides a more specific and reproducible measure of minimal microvascular resistance under conditions of maximal hyperemia (induced by adenosine infusion). Combined interpretation of CFR and IMR enables identification of distinct CMD endotypes (Table 2), including reduced CFR driven by elevated resting flow (functional CMD) and reduced CFR due to impaired hyperemic flow associated with increased minimal resistance (structural CMD), each characterized by different mechanisms and therapeutic implications, although these indices should always be interpreted within the broader clinical and epicardial physiological context [51,57,58,59].

Within this framework, recognizing CMD is essential to define the physiological “ceiling” of epicardial revascularization, prevent inappropriate procedural escalation, and redirect management toward optimized mechanism-based medical therapy and risk-factor modification. This paradigm is particularly relevant in patients with angina and non-obstructive coronary arteries, and remains relevant in the post-PCI setting.

More recently, adenosine-independent approaches for the assessment of coronary microvascular resistance have been developed, including angiography-derived indices and alternative physiological methods that do not require pharmacological hyperemia. Although these techniques may simplify the evaluation of CMD and facilitate the broader adoption of functional testing, further validation and outcome data are required before routine clinical implementation.

In addition to CMD, disorders of coronary vasomotion represent a complementary mechanism of ischemia. Endothelium-dependent vasomotor function can be assessed through intracoronary acetylcholine administration, using lower doses to evaluate endothelial dysfunction and higher doses to provoke epicardial or microvascular spasm. Epicardial or microvascular vasospasm can contribute to anginal symptoms in patients with non-obstructive coronary arteries or after apparently successful revascularization [60,61].

#### 3.2.2. From Selective Protocols to the #FullPhysiology Approach

**Figure 2:**
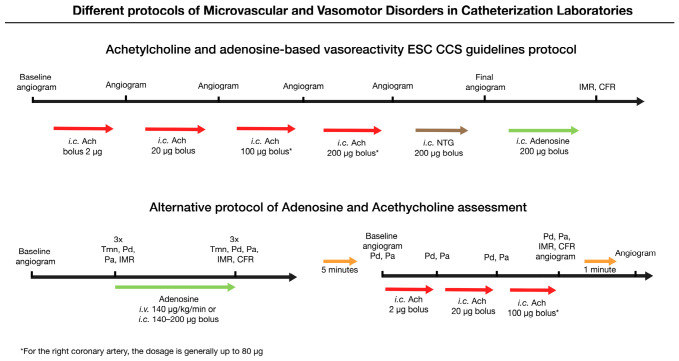
CCS 2024 guideline-based [1] and alternative protocol [62] for the invasive assessment of microvascular and vasomotor disorders in the catheterization laboratory. Ach: Acetylcholine; i.c.: Intracoronary; i.v.: Intravenous; NTG: Nitroglycerin; CFR: Coronary Flow Reserve; IMR: Index of Microcirculatory Resistance; Pd: Distal Coronary Pressure; Pa: Aortic Pressure; Tmn: Mean Transit Time.

Different protocols have been adopted in clinical practice for functional testing of CMD and vasomotor disorders. In ANOCA/INOCA, the 2024 ESC CCS guidelines propose a protocol in which vasoreactivity is assessed first with acetylcholine, followed by evaluation of coronary microvascular function with adenosine [1]. However, alternative approaches have been proposed, such as the protocol developed by Miner et al., in which the adenosine response is assessed first, followed by acetylcholine testing (Figure 2) [62].

Variability also exists in acetylcholine dosing protocols, particularly for right coronary artery testing, reflecting the lack of complete standardization across studies and clinical practice. (Figure 2).

A recent study by Rehan et al. demonstrated that high-dose acetylcholine provocation (200 micrograms for the left coronary artery and 80 micrograms for the right coronary artery) improves the detection of vasomotor disorders in patients with ANOCA, but may increase the risk of overdiagnosis. Therefore, high doses of acetylcholine should be reserved for patients with a high clinical suspicion of vasomotor disorders, and results should be interpreted within the broader clinical context [63].

In response to the need to standardize doses, techniques, and workflow for the assessment of both epicardial and microvascular coronary disease, a comprehensive evaluation strategy has been proposed, culminating in a systematic and personalized approach referred to as #FullPhysiology. This approach encompasses epicardial assessment with resting and hyperemic indices and pressure pullback; microvascular evaluation using thermodilution-derived CFR and IMR; vasomotor testing; and post-PCI physiological reassessment [64].

Although not yet formally incorporated into clinical guidelines, the #FullPhysiology approach represents a pragmatic, evidence-informed framework that is increasingly adopted in contemporary practice. By leveraging wire-based pressure and thermodilution technologies, it enables comprehensive coronary functional phenotyping without substantial increases in procedural time; moreover, available real-world data suggest that the additional upfront costs associated with comprehensive physiological assessment may be offset by reductions in repeat diagnostic testing, rehospitalizations, and unnecessary interventions [65].

#### 3.2.3. Advanced Physiological Endotyping in ANOCA

Recent high-quality evidence demonstrates that ANOCA is not a monolithic entity but rather comprises a spectrum of distinct hemodynamic phenotypes that can be identified through systematic invasive functional testing. In the prospective multicenter study by Miner and colleagues, responses to adenosine and acetylcholine were quantified in over 1000 patients with suspected ANOCA, enabling the definition of eight discrete physiological endotypes based on resting and hyperemic flow characteristics, microvascular resistance, and vasomotor reactivity [62].

The identified endotypes encompassed abnormalities of adenosine-mediated microvascular function, vasomotor disorders, and altered nociception (Table 2). More than one endotype was present in approximately 12% of patients, while nearly one quarter had entirely normal physiological responses despite symptoms [62]. Importantly, each endotype was associated with distinct clinical correlates and, through a three-step Delphi consensus process, specific medical therapies were proposed for all categories. High resting flow and compensated resistance phenotypes may be better addressed with therapies aimed at modulating myocardial oxygen demand and improving microvascular vasodilatory capacity, whereas high-resistance phenotypes may benefit from intensive risk-factor modification and agents targeting microvascular remodeling. Epicardial and microvascular spasm endotypes align with the use of calcium-channel blockers and nitrates, and endothelial dysfunction may respond preferentially to therapies that improve endothelial health, such as statins, ACE inhibitors, and lifestyle-based interventions. By contrast, patients with ischemia in the absence of hemodynamic abnormalities or with enhanced nociception may derive limited benefit from conventional anti-ischemic therapy, pointing instead toward neuromodulatory, rehabilitative, or symptom-directed strategies [62].

However, the proposed treatment strategies are based on expert consensus generated through a Delphi process and remain supported by limited prospective evidence. Consequently, they should be viewed as a framework for individualized management rather than definitive endotype-specific therapeutic recommendations.

This refined endotype classification provides proof of concept that invasive physiology enables mechanism-guided phenotyping and personalized therapy. By linking physiological patterns to targeted treatments, this approach holds promise for improving symptom control and quality of life in patients with ANOCA while minimizing ineffective interventions. The ongoing INOCA-IT multicenter registry (NCT05164640) will evaluate the impact of a stratified diagnostic and therapeutic approach on symptom burden and prognosis in patients with INOCA [66].

#### 3.2.4. Innovative and Emerging Therapeutic Approaches for ANOCA/INOCA

As physiological endotyping evolves, therapeutic strategies increasingly target underlying mechanisms rather than symptoms alone. Among device-based interventions, coronary sinus reduction has emerged as a promising approach for patients with refractory angina and microvascular dysfunction [67]. The multicenter prospective INROAD study reported that implantation of a coronary sinus reducer was associated with improvements in invasive indices of microcirculatory function, including a reduction in IMR and increases in CFR, alongside clinical improvements in angina severity and quality of life four months after the procedure [68]. These findings provide mechanistic insight into how augmenting venous pressure and redistributing myocardial blood flow—particularly toward subendocardial regions—may alleviate ischemia in patients in whom conventional epicardial interventions have limited efficacy [69].

In addition to device-based therapies, regenerative approaches, including autologous CD34^+^ stem cell therapy, are being explored with promising results [70]. Adjunctive non-invasive strategies may also play complementary roles in ANOCA/INOCA management. Structured cardiac rehabilitation and cognitive-behavioral interventions can address functional and psychosocial determinants of symptoms, improve exercise capacity, and enhance overall wellbeing [70].

While many of these approaches remain investigational or are supported by limited data, they reflect a broader trend toward mechanism-tailored therapy in ANOCA/INOCA. Future randomized trials and longer-term outcome studies will be essential to define which patients derive the greatest benefit from each modality and how these therapies can be integrated into physiology-guided care pathways.

## 4. Conclusions

Invasive coronary physiology has evolved from a lesion-selection tool into a comprehensive framework for understanding coronary disease. By integrating pressure-derived indices, longitudinal vessel analysis, microvascular assessment, and vasomotor testing, contemporary physiology enables precise phenotyping of ischemia and more rational use of PCI. A mechanism-based, physiology-guided approach, incorporating continuous assessment of epicardial disease patterns rather than reliance on binary classifications alone, is therefore essential to optimize outcomes, avoid futile interventions, and deliver truly personalized care across the full spectrum of coronary syndromes.

## Figures and Tables

**Table 1:** Pre- and Post-PCI invasive physiology longitudinal vessel analysis.

Pre-PCI Invasive Physiology Vessel Characterization		
**Pattern of CAD**	**Hyperemic measure**	**Non-Hyperemic measure**
**Focal**	FFR ≤ 0.80 with a single abrupt pressure drop	iFR/RFR/dPR ≤ 0.89 with an abrupt decrease ≥0.03 over ≤15 mm.
**Tandem**	FFR ≤ 0.80 with ≥2 abrupt pressure drops	iFR/RFR/dPR ≤ 0.89 with ≥2 discrete index drops
**Diffuse**	FFR ≤ 0.80 with progressive, linear pressure loss along the vessel	iFR/RFR/dPR ≤ 0.89 with gradual pressure decline throughout the pullback
**Post-PCI invasive physiology vessel characterization**		
**Pattern of CAD**	**Hyperemic measure**	**Non-hyperemic measure**
**Focal**	FFR with abrupt pressure loss at or outside the stented segment	Abrupt drop of iFR/RFR/dPR at or outside the stented segment
**Tandem**	Abrupt drop of FFR at an untreated adjacent stenosis	Abrupt drop of iFR/RFR/dPR values at the level of an untreated adjacent stenosis
**Diffuse**	Progressive, linear loss in FFR along the treated vessel	Progressive, linear loss in iFR/RFR/dPR along the treated vessel

PCI: percutaneous coronary intervention, CAD: coronary artery disease, FFR: fractional flow reserve, iFR: instantaneous wave-free ratio, RFR: resting full-cycle ratio, dPR: diastolic pressure ratio.

**Table 2:** Endotypes classification of vasomotor and microvascular disorders.

Abnormal Adenosine Responses	
**High resting coronary blood flow** **(*functional CMD*)**	Adenosine-CFR < 2.5 with adenosine-IMR < 25
**High resistance** **(*structural CMD*)**	Adenosine-CFR < 2.5 with adenosine-IMR > 25
**Compensated high resistance**	Adenosine-CFR > 2.5 with adenosine-IMR > 25
**Abnormal acetylcholine responses**	
**Epicardial spasm**	Acetylcholine-Pd/Pa < 0.80 and/or > 90% coronary diameter reduction with ECG changes and angina during acetylcholine
**Microvascular spasm**	Acetylcholine-IMR > rest-IMR with ECG changes and angina during acetylcholine
**Impaired endothelium- dependent** **vasodilation**	Acetylcholine-CFR < 1.5 with preserved Adenosine-CFR and no microvascular spasm
**Ischemia without hemodynamic** **abnormalities**	Angina AND ischemic ECG changes during acetylcholine without measurable hemodynamic abnormalities
**Altered nociception**	
**Enhanced nociception**	Angina WITHOUT ischemic ECG changes or hemodynamic abnormalities during acetylcholine infusion

CMD: coronary microvascular dysfunction, CFR: coronary flow reserve, IMR: index of microvascular resistance, Pd/Pa: Distal Coronary Pressure to Aortic Pressure Ratio, ECG: electrocardiogram.

## Data Availability

This study is based exclusively on previously published data. No new datasets, materials, or computer code were generated. All referenced studies are publicly available through their respective publishers or databases. No restrictions apply to the availability of the materials or information used in this review.

## Abbreviations

The following abbreviations are used in this manuscript:

ANOCA	Angina with Non-Obstructive Coronary Arteries
AHA	American Heart Association
CAD	Coronary Artery Disease
CCS	Chronic Coronary Syndromes
CFR	Coronary Flow Reserve
CMD	Coronary Microvascular Dysfunction
EAPCI	European Association of Percutaneous Cardiovascular Interventions
ESC	European Society of Cardiology
FFR	Fractional Flow Reserve
IMR	Index of Microcirculatory Resistance
INOCA	Ischemia with Non-Obstructive Coronary Arteries
iFR	Instantaneous Wave-Free Ratio
IVUS	Intravascular Ultrasound
LAD	Left Anterior Descending
MINOCA	Myocardial Infarction with Non-Obstructive Coronary Arteries
NHPRs	Non-Hyperemic Pressure Ratios
OCT	Optical Coherence Tomography
PCI	Percutaneous Coronary Intervention
PPG	Pullback Pressure Gradient
RFR	Resting Full-Cycle Ratio
dPR	Diastolic Pressure Ratio

## References

[1] Vrints C., Andreotti F., Koskinas K.C., Rossello X., Adamo M., Ainslie J., Banning A.P., Budaj A., Buechel R.R., Chiariello G.A. (2024). 2024 ESC Guidelines for the Management of Chronic Coronary Syndromes. Eur. Heart J..

[2] Virani S.S., Newby L.K., Arnold S.V., Bittner V., Brewer L.C., Demeter S.H., Dixon D.L., Fearon W.F., Hess B., Johnson H.M. (2023). 2023 AHA/ACC/ACCP/ASPC/NLA/PCNA Guideline for the Management of Patients with Chronic Coronary Disease: A Report of the American Heart Association/American College of Cardiology Joint Committee on Clinical Practice Guidelines. Circulation.

[3] Tonino P.A.L., De Bruyne B., Pijls N.H.J., Siebert U., Ikeno F., van `t Veer M., Klauss V., Manoharan G., Engstrøm T., Oldroyd K.G. (2009). Fractional Flow Reserve versus Angiography for Guiding Percutaneous Coronary Intervention. N. Engl. J. Med..

[4] De Bruyne B., Pijls N.H.J., Kalesan B., Barbato E., Tonino P.A.L., Piroth Z., Jagic N., Möbius-Winkler S., Rioufol G., Witt N. (2012). Fractional Flow Reserve–Guided PCI versus Medical Therapy in Stable Coronary Disease. N. Engl. J. Med..

[5] Jeremias A., Davies J.E., Maehara A., Matsumura M., Schneider J., Tang K., Talwar S., Marques K., Shammas N.W., Gruberg L. (2019). Blinded Physiological Assessment of Residual Ischemia After Successful Angiographic Percutaneous Coronary Intervention. JACC Cardiovasc. Interv..

[6] Patel M.R., Jeremias A., Maehara A., Matsumura M., Zhang Z., Schneider J., Tang K., Talwar S., Marques K., Shammas N.W. (2022). 1-Year Outcomes of Blinded Physiological Assessment of Residual Ischemia After Successful PCI. JACC Cardiovasc. Interv..

[7] Collison D., Didagelos M., Aetesam-ur-Rahman M., Copt S., McDade R., McCartney P., Ford T.J., McClure J., Lindsay M., Shaukat A. (2021). Post-Stenting Fractional Flow Reserve vs Coronary Angiography for Optimization of Percutaneous Coronary Intervention (TARGET-FFR). Eur. Heart J..

[8] Pijls N.H.J., de Bruyne B., Peels K., van der Voort P.H., Bonnier H.J.R.M., Bartunek J., Koolen J.J. (1996). Measurement of Fractional Flow Reserve to Assess the Functional Severity of Coronary-Artery Stenoses. N. Engl. J. Med..

[9] Kikuta Y., Cook C.M., Sharp A.S.P., Salinas P., Kawase Y., Shiono Y., Giavarini A., Nakayama M., De Rosa S., Sen S. (2018). Pre-Angioplasty Instantaneous Wave-Free Ratio Pullback Predicts Hemodynamic Outcome In Humans with Coronary Artery Disease. JACC Cardiovasc. Interv..

[10] Escaned J., Berry C., De Bruyne B., Shabbir A., Collet C., Lee J.M., Appelman Y., Barbato E., Biscaglia S., Buszman P.P. (2023). Applied Coronary Physiology for Planning and Guidance of Percutaneous Coronary Interventions. A Clinical Consensus Statement from the European Association of Percutaneous Cardiovascular Interventions (EAPCI) of the European Society of Cardiology. EuroIntervention.

[11] Collet C., Sonck J., Vandeloo B., Mizukami T., Roosens B., Lochy S., Argacha J.-F., Schoors D., Colaiori I., Di Gioia G. (2019). Measurement of Hyperemic Pullback Pressure Gradients to Characterize Patterns of Coronary Atherosclerosis. J. Am. Coll. Cardiol..

[12] Fearon W.F., Bornschein B., Tonino P.A.L., Gothe R.M., Bruyne B.D., Pijls N.H.J., Siebert U. (2010). Economic Evaluation of Fractional Flow Reserve–Guided Percutaneous Coronary Intervention in Patients with Multivessel Disease. Circulation.

[13] Fearon W.F., Shilane D., Pijls N.H.J., Boothroyd D.B., Tonino P.A.L., Barbato E., Jüni P., De Bruyne B., Hlatky M.A. (2013). Cost-Effectiveness of Percutaneous Coronary Intervention in Patients with Stable Coronary Artery Disease and Abnormal Fractional Flow Reserve. Circulation.

[14] Fearon W.F., Nishi T., De Bruyne B., Boothroyd D.B., Barbato E., Tonino P., Jüni P., Pijls N.H.J., Hlatky M.A. (2018). Clinical Outcomes and Cost-Effectiveness of Fractional Flow Reserve–Guided Percutaneous Coronary Intervention in Patients with Stable Coronary Artery Disease. Circulation.

[15] Bech G.J.W., De Bruyne B., Pijls N.H.J., de Muinck E.D., Hoorntje J.C.A., Escaned J., Stella P.R., Boersma E., Bartunek J., Koolen J.J. (2001). Fractional Flow Reserve to Determine the Appropriateness of Angioplasty in Moderate Coronary Stenosis. Circulation.

[16] Davies J.E., Sen S., Dehbi H.-M., Al-Lamee R., Petraco R., Nijjer S.S., Bhindi R., Lehman S.J., Walters D., Sapontis J. (2017). Use of the Instantaneous Wave-Free Ratio or Fractional Flow Reserve in PCI. N. Engl. J. Med..

[17] Götberg M., Christiansen E.H., Gudmundsdottir I.J., Sandhall L., Danielewicz M., Jakobsen L., Olsson S.-E., Öhagen P., Olsson H., Omerovic E. (2017). Instantaneous Wave-Free Ratio versus Fractional Flow Reserve to Guide PCI. N. Engl. J. Med..

[18] Berry C., McClure J.D., Oldroyd K.G. (2023). Coronary Revascularization Guided by Instantaneous Wave-Free Ratio Compared with Fractional Flow Reserve: Pooled 5-Year Mortality in the DEFINE-FLAIR and IFR-SWEDEHEART Trials. Eur. Heart J..

[19] Eftekhari A., Holck E.N., Westra J., Olsen N.T., Bruun N.H., Jensen L.O., Engstrøm T., Christiansen E.H. (2023). Instantaneous Wave Free Ratio vs. Fractional Flow Reserve and 5-Year Mortality: IFR SWEDEHEART and DEFINE FLAIR. Eur. Heart J..

[20] De Maria G.L., Garcia-Garcia H.M., Scarsini R., Hideo-Kajita A., Gonzalo López N., Leone A.M., Sarno G., Daemen J., Shlofmitz E., Jeremias A. (2020). Novel Indices of Coronary Physiology. Circ. Cardiovasc. Interv..

[21] Lee S.H., Choi K.H., Lee J.M., Hwang D., Rhee T.-M., Park J., Kim H.K., Cho Y.-K., Yoon H.-J., Park J. (2019). Physiologic Characteristics and Clinical Outcomes of Patients with Discordance Between FFR and IFR. JACC Cardiovasc. Interv..

[22] Jeremias A., Maehara A., Généreux P., Asrress K.N., Berry C., De Bruyne B., Davies J.E., Escaned J., Fearon W.F., Gould K.L. (2014). Multicenter Core Laboratory Comparison of the Instantaneous Wave-Free Ratio and Resting P*_d_*/P*_a_* with Fractional Flow Reserve. J. Am. Coll. Cardiol..

[23] Cook C.M., Jeremias A., Petraco R., Sen S., Nijjer S., Shun-Shin M.J., Ahmad Y., de Waard G., van de Hoef T., Echavarria-Pinto M. (2017). Fractional Flow Reserve/Instantaneous Wave-Free Ratio Discordance in Angiographically Intermediate Coronary Stenoses. JACC Cardiovasc. Interv..

[24] Praz F., Borger M.A., Lanz J., Marin-Cuartas M., Abreu A., Adamo M., Ajmone Marsan N., Barili F., Bonaros N., Cosyns B. (2025). 2025 ESC/EACTS Guidelines for the Management of Valvular Heart Disease. Eur. Heart J..

[25] Byrne R.A., Rossello X., Coughlan J.J., Barbato E., Berry C., Chieffo A., Claeys M.J., Dan G.-A., Dweck M.R., Galbraith M. (2023). 2023 ESC Guidelines for the Management of Acute Coronary Syndromes. Eur. Heart J..

[26] Prati F., Albertucci M., Biccire’ F.G., Gatto L. (2025). The Role of Functional Assessment in the Management of Ischaemic Heart Disease. Eur. Heart J. Suppl..

[27] Escaned J., Petraco R., Fearon W.F. (2024). Coronary Physiology to Guide Percutaneous Coronary Intervention: Why, When, and How. J. Soc. Cardiovasc. Angiogr. Interv..

[28] Bangalore S., Fearon W.F., Fugar S., Dangas G.D., Iqbal S., Johnson N.P., Power D., Tamis-Holland J., Kern M.J. (2025). Evidence-Based Practices in the Cardiac Catheterization Laboratory: Invasive Epicardial Coronary Physiologic Assessment: A Scientific Statement from the American Heart Association. Circulation.

[29] Higashioka D., Shiono Y., Kubo T., Kitabata H., Nishi T., Terada K., Emori H., Takahata M., Wada T., Shimamura K. (2020). The Inter-Study Reproducibility of Instantaneous Wave-Free Ratio and Angiography Coregistration. J. Cardiol..

[30] Perea-Armijo J., Resúa-Collazo A., Herrera-Flores J., Suárez de Lezo J., Ojeda S., Pan-Álvarez M. Diffusely Positive Instantaneous Wave-Free Ratio in the Left Anterior Descending Artery: Long-Term Prognosis with Medical Therapy.

[31] Sonck J., Mizukami T., Johnson N.P., Nagumo S., Gallinoro E., Candreva A., Mileva N., Munhoz D., Shinke T., Svanerud J. (2022). Development, Validation, and Reproducibility of the Pullback Pressure Gradient (PPG) Derived from Manual Fractional Flow Reserve Pullbacks. Catheter. Cardiovasc. Interv..

[32] Carvalho P.E.P., Collet C., De Bruyne B., Munhoz D., Sonck J., Sara J.D.S., Strepkos D., Mutlu D., Alexandrou M., Ser O.S. (2025). The Pullback Pressure Gradient. JACC Adv..

[33] Candreva A., Mizukami T., Sonck J., Munhoz D., Nagumo S., Di Gioia G., Gallinoro E., Mileva N., Bartunek J., Wyffels E. (2021). Hyperemic Hemodynamic Characteristics of Serial Coronary Lesions Assessed by Pullback Pressure Gradients. Catheter. Cardiovasc. Interv..

[34] Ohashi H., Mizukami T., Sonck J., Bouisset F., Ko B., Nørgaard B.L., Mæng M., Jensen J.M., Sakai K., Ando H. (2024). Intravascular Imaging Findings After PCI in Patients with Focal and Diffuse Coronary Artery Disease. J. Am. Heart Assoc..

[35] Mizukami T., Sonck J., Sakai K., Ko B., Maeng M., Otake H., Koo B., Nagumo S., Nørgaard B.L., Leipsic J. (2022). Procedural Outcomes After Percutaneous Coronary Interventions in Focal and Diffuse Coronary Artery Disease. J. Am. Heart Assoc..

[36] De Bruyne B., Pijls N.H.J., Heyndrickx G.R., Hodeige D., Kirkeeide R., Gould K.L. (2000). Pressure-Derived Fractional Flow Reserve to Assess Serial Epicardial Stenoses. Circulation.

[37] Li Kam Wa M.E., Ezad S.M., Modi B., Demir O.M., Hinton J., Ellis H., De Silva K., Gulati A., De Silva R., O’Kane P. (2025). Randomized Comparison of Fractional Flow Reserve and Instantaneous Wave Free Ratio in Serial Disease. JACC Cardiovasc. Interv..

[38] Collet C., Collison D., Mizukami T., McCartney P., Sonck J., Ford T., Munhoz D., Berry C., De Bruyne B., Oldroyd K. (2022). Differential Improvement in Angina and Health-Related Quality of Life After PCI in Focal and Diffuse Coronary Artery Disease. JACC Cardiovasc. Interv..

[39] Collet C., Munhoz D., Mizukami T., Sonck J., Matsuo H., Shinke T., Ando H., Ko B., Biscaglia S., Rivero F. (2024). Influence of Pathophysiologic Patterns of Coronary Artery Disease on Immediate Percutaneous Coronary Intervention Outcomes. Circulation.

[40] Ulacia Flores P., Cieza T., Ouarrak S., Ruhl A., Mengi S., De Larochellière R., Garcia-Labbé D., Déry J.-P., Poulin A., Larose É. (2025). Randomized Study Comparing Angiography Guidance with Physiology Guidance After PCI: The EASY-PREDICT Study. Circ. Cardiovasc. Interv..

[41] Zhang J., Gao X., Kan J., Ge Z., Han L., Lu S., Tian N., Lin S., Lu Q., Wu X. (2018). Intravascular Ultrasound Versus Angiography-Guided Drug-Eluting Stent Implantation. J. Am. Coll. Cardiol..

[42] Hwang D., Lee J.M., Lee H.-J., Kim S.H., Nam C.-W., Hahn J.-Y., Shin E.-S., Matsuo A., Tanaka N., Matsuo H. (2019). Influence of Target Vessel on Prognostic Relevance of Fractional Flow Reserve After Coronary Stenting. EuroIntervention.

[43] Collet C., Johnson N.P., Mizukami T., Fearon W.F., Berry C., Sonck J., Collison D., Koo B.-K., Meneveau N., Agarwal S.K. (2023). Impact of Post-PCI FFR Stratified by Coronary Artery. JACC Cardiovasc. Interv..

[44] Räber L., Mintz G.S., Koskinas K.C., Johnson T.W., Holm N.R., Onuma Y., Radu M.D., Joner M., Yu B., Jia H. (2018). Clinical Use of Intracoronary Imaging. Part 1: Guidance and Optimization of Coronary Interventions. An Expert Consensus Document of the European Association of Percutaneous Cardiovascular Interventions. Eur. Heart J..

[45] Meneveau N., Souteyrand G., Motreff P., Caussin C., Amabile N., Ohlmann P., Morel O., Lefrançois Y., Descotes-Genon V., Silvain J. (2016). Optical Coherence Tomography to Optimize Results of Percutaneous Coronary Intervention in Patients with Non–ST-Elevation Acute Coronary Syndrome. Circulation.

[46] Mandurino-Mirizzi A., Munafò A.R., Rizzo F., Raone L., Germinal F., Montalto C., Mussardo M., Vergallo R., Fischetti D., Godino C. (2025). Comparison of Different Guidance Strategies to Percutaneous Coronary Intervention: A Network Meta-Analysis of Randomized Clinical Trials. Int. J. Cardiol..

[47] Wijns W., Shite J., Jones M.R., Lee S.W.-L., Price M.J., Fabbiocchi F., Barbato E., Akasaka T., Bezerra H., Holmes D. (2015). Optical Coherence Tomography Imaging During Percutaneous Coronary Intervention Impacts Physician Decision-Making: ILUMIEN I Study. Eur. Heart J..

[48] Neleman T., van Zandvoort L.J.C., Tovar Forero M.N., Masdjedi K., Ligthart J.M.R., Witberg K.T., Groenland F.T.W., Cummins P., Lenzen M.J., Boersma E. (2022). FFR-Guided PCI Optimization Directed by High-Definition IVUS Versus Standard of Care. JACC Cardiovasc. Interv..

[49] Buonpane A., Trimarchi G., Di Muro F.M., Nardi G., Ciardetti M., Coceani M.A., Pastormerlo L.E., Paradossi U., Berti S., Trani C. (2025). From Vision to Illumination: The Promethean Journey of Optical Coherence Tomography in Cardiology. J. Clin. Med..

[50] Mensah G.A., Fuster V., Murray C.J.L., Roth G.A., Mensah G.A., Abate Y.H., Abbasian M., Abd-Allah F., Abdollahi A., Abdollahi M. (2023). Global Burden of Cardiovascular Diseases and Risks, 1990–2022. J. Am. Coll. Cardiol..

[51] Hansen B., Holtzman J.N., Juszczynski C., Khan N., Kaur G., Varma B., Gulati M. (2023). Ischemia with No Obstructive Arteries (INOCA): A Review of the Prevalence, Diagnosis and Management. Curr. Probl. Cardiol..

[52] Bairey Merz C.N., Pepine C.J., Walsh M.N., Fleg J.L., Camici P.G., Chilian W.M., Clayton J.A., Cooper L.S., Crea F., Di Carli M. (2017). Ischemia and No Obstructive Coronary Artery Disease (INOCA). Circulation.

[53] Camici P.G., Crea F. (2007). Coronary Microvascular Dysfunction. N. Engl. J. Med..

[54] Ong P., Camici P.G., Beltrame J.F., Crea F., Shimokawa H., Sechtem U., Kaski J.C., Bairey Merz C.N. (2018). International Standardization of Diagnostic Criteria for Microvascular Angina. Int. J. Cardiol..

[55] Ford T.J., Stanley B., Good R., Rocchiccioli P., McEntegart M., Watkins S., Eteiba H., Shaukat A., Lindsay M., Robertson K. (2018). Stratified Medical Therapy Using Invasive Coronary Function Testing in Angina. J. Am. Coll. Cardiol..

[56] Tamis-Holland J.E., Jneid H., Reynolds H.R., Agewall S., Brilakis E.S., Brown T.M., Lerman A., Cushman M., Kumbhani D.J., Arslanian-Engoren C. (2019). Contemporary Diagnosis and Management of Patients with Myocardial Infarction in the Absence of Obstructive Coronary Artery Disease: A Scientific Statement From the American Heart Association. Circulation.

[57] Rahman H., Demir O.M., Khan F., Ryan M., Ellis H., Mills M.T., Chiribiri A., Webb A., Perera D. (2020). Physiological Stratification of Patients with Angina Due to Coronary Microvascular Dysfunction. J. Am. Coll. Cardiol..

[58] Taqueti V.R., Di Carli M.F. (2018). Coronary Microvascular Disease Pathogenic Mechanisms and Therapeutic Options. J. Am. Coll. Cardiol..

[59] Fearon W.F., Balsam L.B., Farouque H.M.O., Robbins R.C., Fitzgerald P.J., Yock P.G., Yeung A.C. (2003). Novel Index for Invasively Assessing the Coronary Microcirculation. Circulation.

[60] Biscaglia S., Uretsky B., Barbato E., Collet C., Onuma Y., Jeremias A., Tebaldi M., Hakeem A., Kogame N., Sonck J. (2021). Invasive Coronary Physiology After Stent Implantation. JACC Cardiovasc. Interv..

[61] Ford T.J., Ong P., Sechtem U., Beltrame J., Camici P.G., Crea F., Kaski J.-C., Bairey Merz C.N., Pepine C.J., Shimokawa H. (2020). Assessment of Vascular Dysfunction in Patients Without Obstructive Coronary Artery Disease. JACC Cardiovasc. Interv..

[62] Miner S., Mejia-Renteria H., Leone A.M., Velollari O., Sykes R., Biscaglia S., Esposito G., Galante D., Oreglia J., Ang D. (2025). Endotypes of Angina with Non-Obstructive Coronary Arteries: A Prospective Multicentre Study. Eur. Heart J..

[63] Rehan R., Khandkar C., Wong C.C.Y., Weaver J., Jain P., Adams M., Ng M.K.C., Tremmel J.A., Yong A.S.C. (2025). Diagnostic Validity of Acetylcholine Provocation Protocols in the Evaluation of Coronary Artery Spasm in Patients with ANOCA. Circ. Cardiovasc. Interv..

[64] Scarsini R., Campo G., Di Serafino L., Zanon S., Rubino F., Monizzi G., Biscaglia S., Ancona M., Polimeni A., Niccoli G. (2023). #FullPhysiology: A Systematic Step-by-Step Guide to Implement Intracoronary Physiology in Daily Practice. Minerva Cardiol. Angiol..

[65] Leone A.M., Galante D., Viceré A., Marrone A., Verardi F.M., Giuliana C., Pollio Benvenuto C., Viccaro V., Todisco S., Erriquez A. (2025). Functional Coronary Assessment in Angina with Intermediate Coronary Stenosis: The #FullPhysiology Approach. Eur. Heart J..

[66] Ghizzoni G., Leone A.M., di Serafino L., Galante D., Esposito G., Montorfano M., Chieffo A. (2024). The INOCA-IT: Rationale and Design of a Multicenter Registry Investigating Ischemia in Patients with Non-Obstructive Coronary Artery (INOCA) Disease in Italy. Int. J. Cardiol..

[67] Caffè A., Montone R.A. (2025). Device-Based Therapies for Refractory Angina. J. Clin. Med..

[68] Tebaldi M., Campo G., Ugo F., Guarracini S., Marrone A., Clò S., Abdirashid M., Di Mauro M., Rametta F., Di Marco M. (2024). Coronary Sinus Narrowing Improves Coronary Microcirculation Function in Patients with Refractory Angina: A Multicenter Prospective INROAD Study. Circ. Cardiovasc. Interv..

[69] Henry T.D., Bairey Merz C.N., Wei J., Corban M.T., Quesada O., Joung S., Kotynski C.L., Wang J., Lewis M., Schumacher A.M. (2022). Autologous CD34+ Stem Cell Therapy Increases Coronary Flow Reserve and Reduces Angina in Patients with Coronary Microvascular Dysfunction. Circ. Cardiovasc. Interv..

[70] Gurgoglione F.L., Benatti G., Denegri A., Donelli D., Covani M., De Gregorio M., Dallaglio G., Navacchi R., Niccoli G. (2025). Coronary Microvascular Dysfunction: Insights on Prognosis and Future Perspectives. Rev. Cardiovasc. Med..

